# Continuous-Time Laser Frames Associating and Mapping via Multilayer Optimization

**DOI:** 10.3390/s21010097

**Published:** 2020-12-25

**Authors:** Shaoxing Hu, Shen Xiao, Aiwu Zhang, Yiming Deng, Bingke Wang

**Affiliations:** 1 School of Mechanical Engineering and Automation, Beihang University, Beijing 100191, China; zy1707502@buaa.edu.cn (S.X.); 1920wangbk@buaa.edu.cn (B.W.); 2 Key Laboratory of 3D Information Acquisition and Application, Ministry of Education, Capital Normal University, Beijing 100048, China; 3 Center for Geographic Environment Research and Education, Capital Normal University, Beijing 100048, China; 4 Nondestructive Evaluation Laboratory, Department of Electrical and Computer Engineering of the College of Engineering, Michigan State University, East Lansing, MI 48824, USA; dengyimi@egr.msu.edu

**Keywords:** SLAM, multilayer optimization, interframe association, submap matching, loop detection

## Abstract

To achieve the ability of associating continuous-time laser frames is of vital importance but challenging for hand-held or backpack simultaneous localization and mapping (SLAM). In this study, the complex associating and mapping problem is investigated and modeled as a multilayer optimization problem to realize low drift localization and point cloud map reconstruction without the assistance of the GNSS/INS navigation systems. 3D point clouds are aligned among consecutive frames, submaps, and closed-loop frames using the normal distributions transform (NDT) algorithm and the iterative closest point (ICP) algorithm. The ground points are extracted automatically, while the non-ground points are automatically segmented to different point clusters with some noise point clusters omitted before 3D point clouds are aligned. Through the three levels of interframe association, submap matching and closed-loop optimization, the continuous-time laser frames can be accurately associated to guarantee the consistency of 3D point cloud map. Finally, the proposed method was evaluated in different scenarios, the experimental results showed that the proposed method could not only achieve accurate mapping even in the complex scenes, but also successfully handle sparse laser frames well, which is critical for the scanners such as the new Velodyne VLP-16 scanner’s performance.

## 1. Introduction

In recent years, the Velodyne VLP-16 scanner with 16 laser channels has become the core component of many SLAM systems, thus more attention to the point cloud association of the continuous-time laser frames for the Velodyne VLP-16 was drawn. However, the point clouds from this scanner are sparser, and the association of continuous-time sparser laser frames without the assistance of other sensors to generate a 3D point cloud map with consistency is really important but remains challenging. The identified key challenges and research questions include: (1) how to select the most appropriate association method to align these sparse point clouds; (2) how to remove the noise point clusters to reduce distractions of the laser frame associating and mapping and (3) how to translate the complex associating and mapping problem to a multilayer optimization problem and guarantee the consistency of 3D point cloud map without point cloud ghosting. 

The interframe point cloud association method is usually based on the iterative closest point (ICP) algorithm [1,2], fast point feature histogram (FPFH) algorithm [3], the global search Super-4PCS algorithm [4] and normal distributions transform (NDT) algorithm [5,6]. The ICP algorithm is widely used, but it is easy to fall into the local optimum; The FPFH algorithm is improved compared to the ICP algorithm, but suffers from expensive computational costs; The Super-4PCS algorithm is based on a global search strategy with a random sample consensus (RANSAC) algorithm, which is costly in computation but has higher association accuracy. The NDT algorithm is the fastest and relatively reliable method among the four algorithms. A comparative analysis of four association methods is shown in Section 3.2.

Each frame of the point cloud data contains ground points, non-ground points and interfering target points, such as leaves, grass, outliers, etc., thus it is necessary to segment point clouds accurately so the interfering targets can be removed. Many interframe association algorithms [7,8,9,10,11,12] would divide the point cloud of each frame into ground points and non-ground points first. In general, ground point data have the following properties: (1) ground points belong to planes, which can be described by mathematical models; (2) it is generally believed that the cloud points with the lowest height value in the point cloud data could belong to the ground. According to these properties, Thrun et al. [7] proposed a grid-based ground segmentation algorithm to separate ground points according to the height information. However, relying only on height segmentation can easily lead to undersegmentation or oversegmentation. Tse [8] used Markov random field (MRF) to improve the accuracy of ground point segmentation. Himmelsbach et al. [9] processed the point cloud data in the polar coordinate system, divided the grid map described by the polar coordinate into different fan-shaped areas, established the ground model of each fan-shaped area, and separated the ground points. Huangfu et al. [10] calculated the normal vector and curvature through continuous iteration, and segmented the ground points. Moosmann et al. [11] distinguished the ground points and non-ground points according to a certain slope threshold. Douillard et al. [12] used the Gaussian process iteration method to extract ground points. The above methods often could lead to undersegmentation or oversegmentation [7], while suffering from expensive computation costs [8,9,10,11,12].

Velodyne VLP-16 scanner belongs to three-dimensional LIDAR, and this work focuses on the development of 3D point cloud data segmentation algorithms. In reference [13], after removing the ground points, the different nearest neighbor algorithms are adopted to segment the point cloud, which is costly in computation. In reference [14], by projecting 3D point cloud data into 2D mesh for segmentation, the algorithm is fast and real-time, but it causes undersegmentation. Moosmann et al. [11] used range images to segment point cloud according to the local convexity of the point cloud. Bogoslavskyi and Stachniss proposed fast range image-based segmentation of sparse 3D laser scans for the online operation method [15]. Although there are misclassification cases, according to the range image, the algorithm is simple and fast, and meets the real-time processing requirements.

As the number of associated frames increases, the cumulative errors are inevitable. In the past, the extended Kalman filter was often used to optimize in order to reduce the cumulative errors, but the algorithm is complicated. The optimization algorithm based on graph theory is efficient and easy to be understood. In recent years, it has become the mainstream algorithm for SLAM back-end optimization. The literatures [16,17] have adopted hierarchical optimization to reduce the cumulative errors. Hierarchical optimization is an easy-to-understand graph optimization method through multiple levels of constraints to adapt to more applications. For examples, a robust pose graph approach for city scale LIDAR mapping method was proposed in [18], which aligned multiple frames into a submap, and combined the multiple submaps into a transaction. A portable 3D LIDAR-based system for the long-term and wide-area people behavior measurement method was introduced in [19], which used the ground plane to restrain the elevation errors in the flat indoor environments.

The complex associating and mapping problem is investigated and modeled as a multilayer optimization problem to realize low drift localization and point cloud map reconstruction without the assistance of the GNSS/INS navigation systems.

In this paper, our aim is to realize low drift localization and point cloud map reconstruction just relying on a Velodyne VLP-16 scanner. A lidar SLAM system (named ASQWolf) developed by our research group is used to capture the consecutive laser frames. The core component of the ASQWolf is a Velodyne VLP-16 scanner, which is installed horizontally in the ASQWolf system. This study mainly solves the problem of continuous-time laser frames associating and mapping without other auxiliary sensors. Therefore, this study designed a novel method to associate 3D point cloud frames via three-layer graph optimization. The first layer is the interframe association, *n* consecutive laser frames are quickly aligned into a submap by using the NDT algorithm. The second layer is submap matching, the ICP registration algorithm is used to match consecutive submaps. The third layer is closed-loop graph optimization, it is used to reduce the cumulative errors of continuous-time laser frame association. The noise point clusters include some scattered points and small point clusters of leaves, small moving targets, etc. [20]. The laser frames from Velodyne VLP -16 scanner are sparse and the ground points and non-ground points (excluding the aforementioned noise point clusters) of consecutive frames are fed into the interframe association layer to be executed for the point cloud registration calculation. Considering the limited amount of computing resources, only the non-ground points (excluding the noise point clusters) of consecutive key frames are chosen to participate in the association calculation between submaps. Finally, the closed-loop graph optimization method is used to reduce the cumulative errors of continuous-time frame association. The novelty and contribution of this study include: (1) ground point extraction, how to classify the original point cloud into ground points and non-ground points; (2) non-ground point subdivision, how to remove the influence of the noise point clusters on continuous-time laser frame association and (3) three-layer graph optimization, how to associate continuous-time frame point clouds and realize low drift localization and point cloud map reconstruction without any other sensors through the three levels of point cloud interframe association, submap matching and closed-loop optimization.

## 2. Materials and Methods

### 2.1. Data Model and System Overview

**Figure 1:**
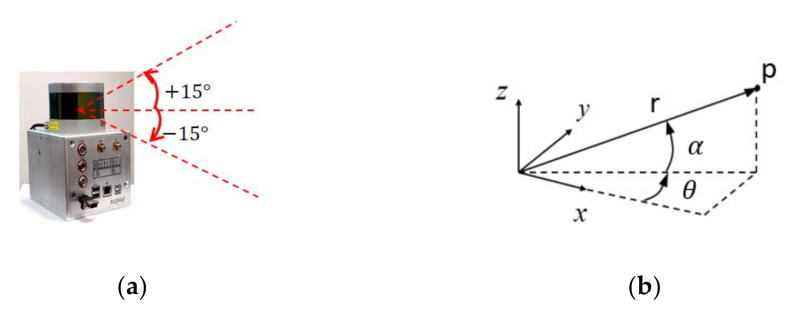
The ASQWolf system with the Velodyne VLP-16 scanner. (**a**) The ASQWolf is handheld to obtain data and (**b**) the coordinate transform is from the cylindrical coordinate system to the rectangular coordinate system.

The ASQWolf, which is a mobile LIDAR instantaneous three-dimensional imaging system developed by our research group, is shown in Figure 1. The Velodyne VLP-16 scanner is one core component of the system, which is installed horizontally in the ASQWolf system. The measurement range of the VLP-16 scanner is up to 100 m with an accuracy of ±3 cm, a vertical field of view (FOV) of ±15° and a horizontal FOV of 360°. The vertical resolution was set as 2°, the horizontal resolution was set as 0.2° and the sampling rate was 10 Hz. The ASQWolf system can be handheld, backpacked or placed on mobile platforms such as robots and cars. This study only discusses the problem of continuous-time laser frames associating and mapping without other auxiliary sensors, so it is not authors’ intention to discuss IMU and other components of the ASQWolf system in this paper. More information of the system can be found in ref [21]. 

Each laser frame acquired by the Velodyne VLP-16 scanner can be arranged according to 1800 horizontal points and 16 vertical points, which are described in the spherical coordinate system (as shown in Figure 1). Each target data point is expressed as $p\left(r,\alpha ,\theta \right)$, where *r* is the distance from the target point to the center of the sensor, α is the pitch angle of the laser beam and θ is the azimuth angle of the laser beam. A range image with 1800 × 16 points was formed, which is expressed as $p\left(i,j\right),\;i\in \left(1,1800\right),\;j\in \left(1,16\right)$. The transform from the spherical coordinate system to the rectangular coordinate system is as follows:

(1) $$\left\{\begin{array}{c} x=rcos\alpha cos\theta \\ y=rcos\alpha sin\theta \\ z=rsin\alpha \;\;\;\;\;\;\;\;\; \end{array}\right.$$

where *x*, *y*, *z* are the 3D coordinates of point *p* in the rectangular coordinate system.

**Figure 2:**
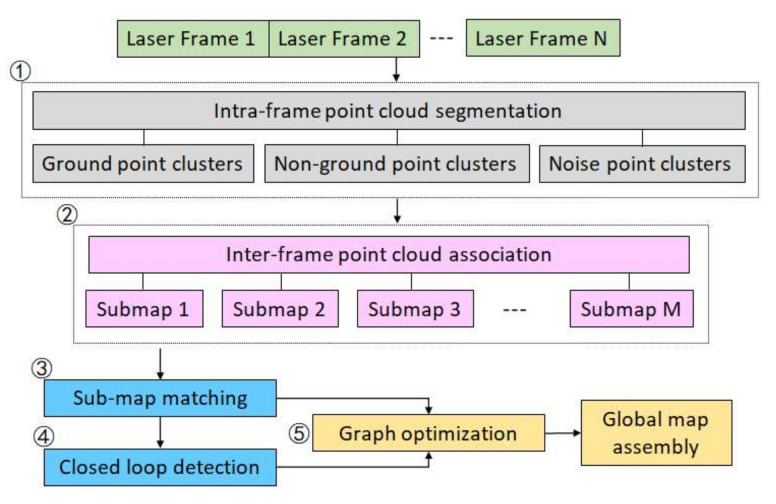
The framework of proposed continuous-time laser frames associating and mapping via multilayer optimization.

Figure 2 describes the technical framework of continuous-time laser frames associating and mapping in this paper. The lidar odometry and mapping (LOAM) algorithm [22] uses features to align consecutive frames, but sometimes the point clouds with few features cannot be aligned. The hdl_graph_slam algorithm [19] uses the NDT algorithm to match the point clouds with a global map, which verifies that the NDT algorithm is suitable for interframe association of sparse point clouds. This study proposed a three-layer continuous-time laser frame association algorithm based on the graph optimization. The algorithm is divided into five main steps: (1) intraframe point cloud segmentation, firstly the ground points are extracted by using random sample consensus (RANSAC) algorithm from continuous-time laser frames with 10 Hz frequency; then, according to the data model of Velodyne VLP-16 horizontal scanning, a range image of 1800×16 points was generated, the non-ground points were subdivided by the method proposed in [15]; finally the noise point clusters with less than 30 points were removed based on the rule mentioned in [20] from the non-ground points. The non-ground points except the noise point clusters were named the effective non-ground points. (2) Interframe point cloud association, where the NDT algorithm is applied to match the consecutive frames with the ground points and effective non-ground points. (3) Submap matching, where a submap is formed after *n* frames of point clouds are aligned, and the first frame of the submap is used as a key frame. The ICP algorithm is applied to match with the key frames with only the effective non-ground points between the submaps. (4) Closed loop detection: when the sensor goes to the position *P_k+m_* from the position $P_{k}\;$after *m* frames, if the point cloud $PC_{k}$ and the point cloud $PC_{k+m}$ are very close and almost coincide, *P_k+m_* and $P_{k}$ forms a closed loop. This study still used the NDT algorithm to quickly detect the closed loop. (5) Graph optimization: the cumulative errors are inevitable for the point cloud association between consecutive frames. In this paper, the key frame was used as the node of the pose graph to perform the optimization adjustment. Finally, the 3D point cloud map was assembled. 

### 2.2. Intraframe Point Cloud Segmentation

#### 2.2.1. Ground Point Extraction

This study used RANSAC algorithm to extract ground points. RANSAC is a commonly used plane fitting method. However, the RANSAC algorithm usually selects points randomly, with many iterations and long calculation time. In this paper, we improved the selecting point method to reduce the running time. Firstly, sort all points of each frame according to the size of their z-coordinates from smallest to largest to obtain the sorted point cloud $P_{sorted}$. Select first $N_{av}$ points from $P_{sorted}$*,* and calculate the average value $Z_{av}$ of their z-coordinates. Choose these points whose z-coordinate are less than $Z_{av}$ as the ground initial seed points. The algorithm is shown in Table 1.

Then, the ground plane was fitted by singular value decomposition (SVD) from the selected initial seed $P_{seed}$, the orthogonal distance from the point to the ground plane was calculated, if the orthogonal distance was less than the set threshold $Th_{dist}$, it is marked as the ground point, otherwise it is marked as the non-ground point. Its algorithm is shown in Table 2.

#### 2.2.2. Remove Interfering Points from Non-Ground Points

Through further subdivision of non-ground points, the noise point clusters, such as some scattered points and small point clusters due to leaves, grass, etc., are removed. Bogoslavskyi and Stachniss proposed the fast range image-based segmentation of sparse 3D laser scans for the online operation method [15], this method could subdivide the non-ground points into some point clusters efficiently. 

**Figure 3:**
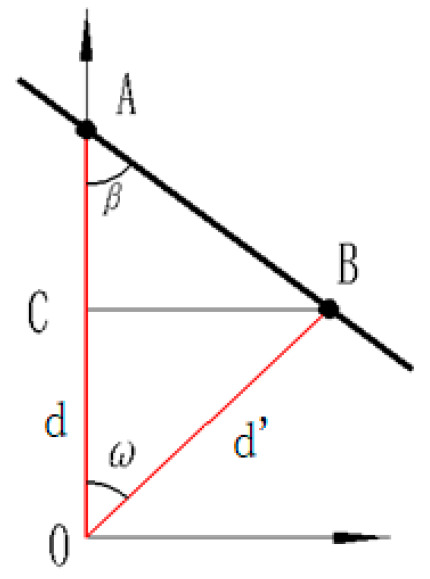
The non-ground point segmentation model based on the range image method [15].

The range image segmentation principle of this method [15] is shown in Figure 3. ω is the angle of the adjacent scan lines, the angle in VLP-16 ω = 0.2°, OA and OB are the laser beams, A and B are two arbitrary points measured from the scanner, the angle β is the angle between the AB line and the laser beam OA. The angle β can be calculated based on the two distances from the center of the sensor to the measured points (A and B). The distance from the sensor center O to the target point A can be represented by d, that is d=‖OA‖. The distance from the sensor center O to the target point B is represented by d′, that is d′=‖OB‖, the angle β can be expressed as:

(2) $$\beta =arctan\frac{\left\|BC\right\|}{\left\|CA\right\|}=arctan\frac{d’sin\omega }{d-d’cos\omega }$$

According to the angle β, determine whether the target points scanned by the adjacent laser scan beams are on the same object, and set the threshold angle θ. If β>θ, they are on the same object, otherwise, they are on the different object.

The non-ground point cloud of the frame at time t is firstly formed into a range image $p_{t}\left(i,j\right),\;i\in \left(1,1800\right),\;j\in \left(1,16\right)$, and then the range image is segmented into many point clusters by the method proposed in [15]. In the ASQWolf system, the vertical resolution and the horizontal resolution of Velodyne VLP-16 scanner are both set as two fixed values. According to the method proposed in [20], the threshold theta to remove interfering points is also set as a fixed value. Some small point clusters with less than 30 points are generated by noise and some small targets such as leaves, grass, small moving targets, etc. The small point clusters were considered as noise point clusters and removed, and then some big point clusters were kept for continuous-time laser frame association. 

### 2.3. Continuous-Time Laser Frame Association

#### 2.3.1. Three-Layer Point Cloud Association

**Figure 4:**
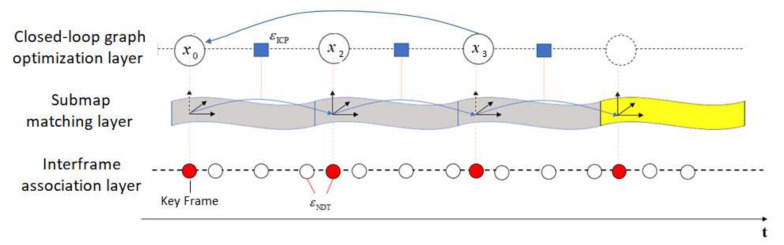
Framework of the three-layer point cloud association technique. In the interframe association layer, the red points represent key frames; in the submap matching layer, the grey submaps denote that they have been matched, the yellow submaps are waiting for matching and in closed-loop graph optimization layer, $x_{i}$ denotes the pose of the *i*-th key frame.

As shown in Figure 4, the first layer is where 3D point clouds are aligned between consecutive frames, which is called the interframe association layer. Several papers [23,24] used the ICP algorithm to align the point clouds. However, if the point cloud of each frame is sparse, and it is difficult to find adequate matching features, the ICP algorithm will easily lead to false results being trapped in the local optimum. Therefore, this study applied the NDT algorithm to match the point clouds between consecutive frames, and form the submaps including *n* frames. In this study, the sampling rate of the ASQWolf system was 10 Hz, i.e., 10 laser frames were obtained in one second, thus, it is reasonable to consider that there was no cumulative error in the submap. 

The second layer is where 3D point clouds are aligned between consecutive submaps, which is called the submap matching layer. The first frame of each submap was used as the key frame, and 3D point clouds of the key frames were aligned between consecutive submaps by the ICP algorithm. For this layer, the key frame was used as a node of the pose graph. Only the key frames were matched in order to reduce the amount of calculation and increase the speed of calculation. In [18], the submap is adaptively constructed based on the pose of each frame. In theory, this adaptive submap construction is more reasonable, but actually increases the computational costs and computing time. Therefore, this study forms the submap according to the number of frames contained in the submap. The number of frames contained in the submap should be different in different environments such as indoors, outdoors and in the forest. It is necessary to determine the number of frames included in the submap through experiments in advance. 

The third layer is the closed-loop graph optimization layer. The pose graph was constructed based on the key frames of the submaps, and the optimization method was performed every time a closed loop was detected. See Section 2.3.2 for details.

#### 2.3.2. Closed-Loop Graph Optimization

After interframe association and submap matching, there were inevitable cumulative errors in the point cloud map, the method of graph optimization was used to further eliminate the cumulative errors. According to the characteristics of the algorithm in this paper, a pose graph was constructed. Following [19], Let $x_{i}$ be the node i of the graph, corresponding to the pose vertex $x_{i}$ of the *i*-th key frame. Let $z_{ij}$ be the edge of the graph, corresponding to the observed values $z_{ij}$ of the pose relationship between the *i*-th key frame and the *j*-th key frame. Following [19,25,26], graph optimization is to minimize the objective function $F\left(x\right)$.

(3) $$F\left(x\right)=\sum_{i,j\in KEY}e{\left(x_{i},x_{j},z_{ij}\right)}^{T}\Omega_{i,j}e\left(x_{i},x_{j},z_{ij}\right),\;x^{*}=\underset{x}{argmin}F\left(x\right)$$

where $x={\left(x_{1},\cdots ,x_{n}\right)}^{T}$is the key frame vector, $x_{i}$,${\;x}_{j}$ corresponds to the pose of the key frame, $z_{ij}$ and $\Omega_{i,j}$ are respectively the observed value of the pose relationship and the information matrix between the *i*-th key frame and the *j*-th key frame, they are considered as the constraints relating $x_{i}$ and $x_{j}$. ${\hat{z}}_{ij}$ represents the prediction of a virtual measurement between the *i*-th key frame and the *j*-th key frame, $e\left(x_{i},x_{j},z_{ij}\right)$ is the error function:

(4) $$e\left(x_{i},x_{j},z_{ij}\right)=z_{ij}-{\hat{z}}_{ij}\left(x_{i},x_{j}\right)$$

Every time a closed loop is detected, the pose graph is updated once with g2o [25] by minimizing the formula$\left(x\right)$.

Closed-loop detection is an important part of the SLAM system and plays a very important role in eliminating accumulated errors. There are many references to the closed-loop detection, for example [27,28]. Considering the detection speed, this paper adopted the fast closed-loop detection method mentioned in the literature [19]. If the following three conditions are satisfied, a closed-loop can be found:

(1) Calculate the distance between the current key frame and the previous key frames, and check whether the translation distance between the two key frames $P_{i}$ to $P_{j}$ is less than the given threshold value (set to 2 m in this paper)
(2) Check whether the length of the sensor trajectory from $P_{i}$ to $P_{j}$ is greater than the given threshold value (set to 10 m in this paper)
(3) If (1) and (2) are both satisfied, the NDT algorithm is used to calculate the fitting degree between $P_{i}$ and $P_{j}$ and determine whether the fitting degree is less than a given threshold value.

## 3. Results

Our approach was evaluated on the raw datasets captured by the ASQWolf system, where we held the ASQWolf system to collect the point clouds while walking. The recording scenes include indoor and outdoor. All data processing and implementation were performed on an Intel i7-8700h PC with 16 GB RAM.

### 3.1. Ground Point Extraction

**Figure 5:**
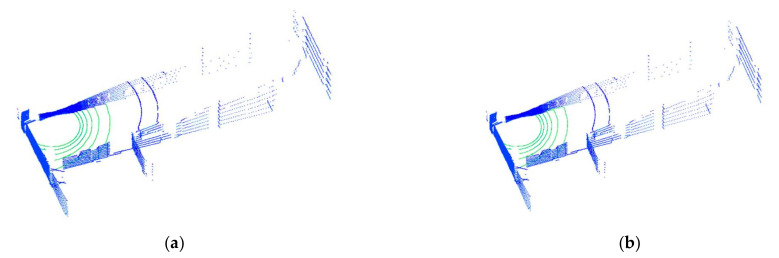

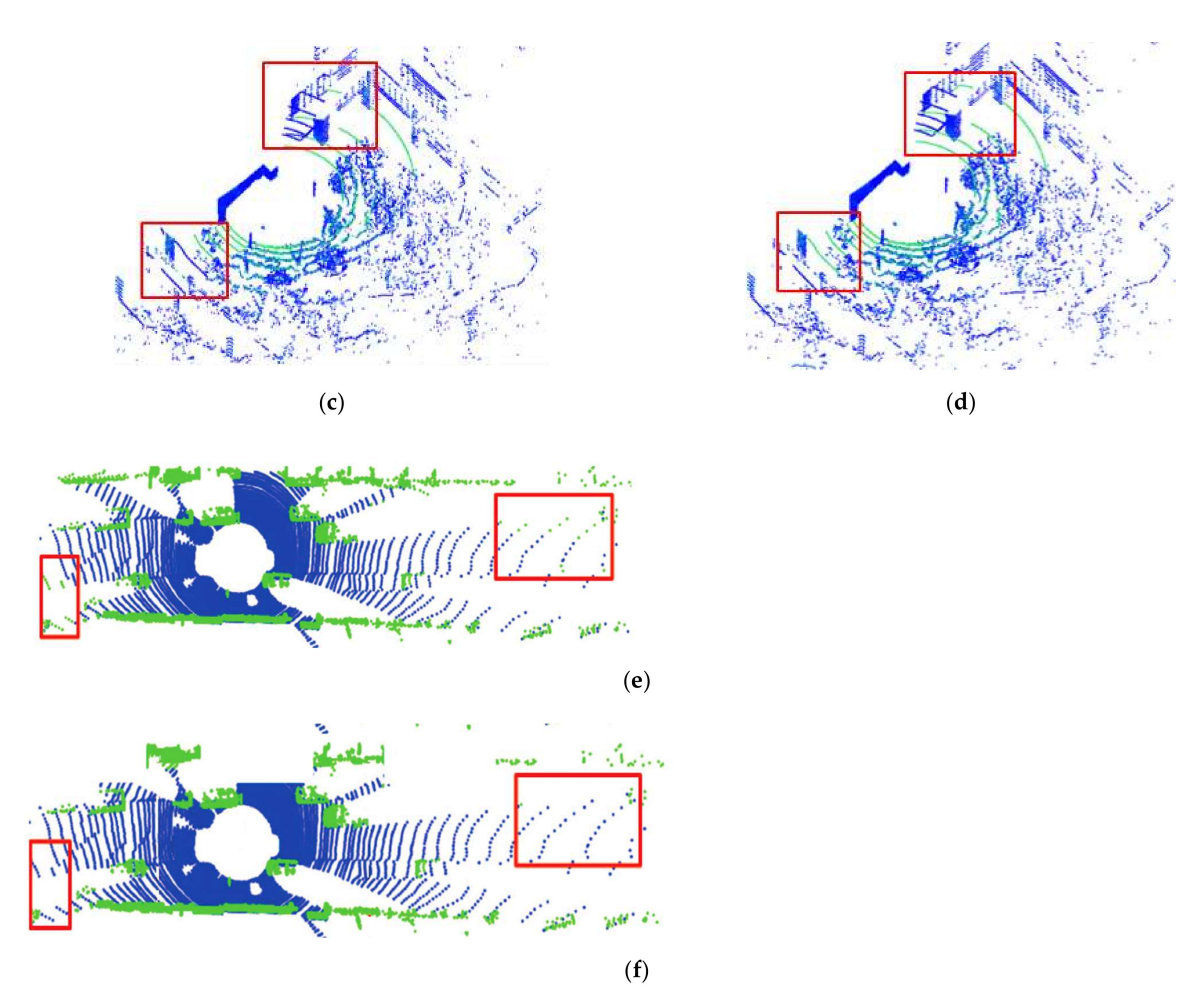
Ground segmentation results of different scenes. The green points are the ground points extracted by the RANSAC algorithm in (**a**,**c**,**e**); the green points are the ground points extracted by the improved RANSAC algorithm in (**b**,**d**,**f**).

Firstly, we compared the accuracy of ground segmentation between the traditional RANSAC algorithm and the improved RANSAC algorithm. The ASQWolf system was used to obtain the point clouds of structured, semistructured and unstructured scenes. The ground points were then extracted from the point clouds of different scenes by the traditional RANSAC algorithm and the improved RANSAC algorithm. Results are shown in Figure 5, where the blue points represent non-ground points, and the green points represent ground points. The threshold $Th_{dist}$ is a key parameter for extracting ground points. In this study, $Th_{dist}$ was set as $Th_{dist}=0.2\;m$ after many tests. In further research, the threshold $Th_{dist}$ will be set as an adaptive threshold for better avoiding undersegmentation or oversegmentation. 

Figure 5a,b are the point clouds of the indoor scene with the structured features. Figure 5c,d are the point clouds of the outdoor scene with semi-structured features. Figure 5e,f are the point clouds of the road area with unstructured features. Figure 5a,c,e are the results of ground point segmentation using the RANSAC algorithm directly, and Figure 5b,d,f are the results of ground point segmentation using the improved RANSAC algorithm. It can be seen from the red box in Figure 5: (1) by using the traditional RANSAC algorithm, the segmentation results of the structured scenes are satisfactory, and the segmentation results of the semistructured and unstructured scenes are not satisfactory and (2) by using the improved RANSAC algorithm, the segmentation results of the structured, semistructured and unstructured scenes are satisfactory.

**Figure 6:**
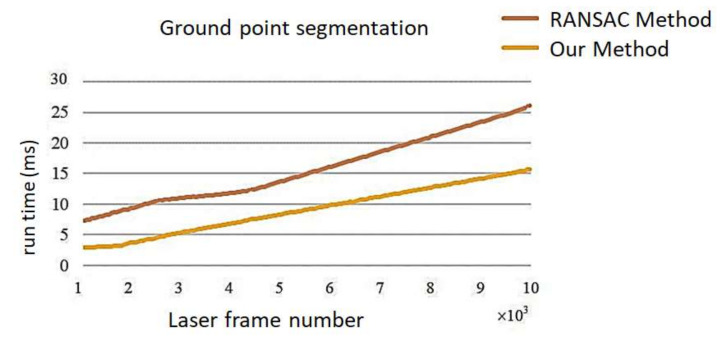
Comparison of ground point segmentation speed.

Secondly, we compared the speed of the ground segmentation between the traditional RANSAC algorithm and the improved RANSAC algorithm. The comparison results are shown in Figure 6, where the *x*-axis is the point number of the point cloud segmented by the two methods, and the *y*-axis is the segmentation time. It shows that the speed of the ground segmentation using the improved RANSAC algorithm is faster that that using the traditional RANSAC algorithm. 

### 3.2. Comparative Analysis of Four Association Methods

In terms of the translation errors, rotation errors and computing time of the point cloud registration, this study compared and analyzed the following four methods: the ICP algorithm based on points, the FPFH algorithm based on feature descriptions, the Super-4PCS algorithm based on global search and the NDT algorithm based on normal distribution.

**Figure 7:**
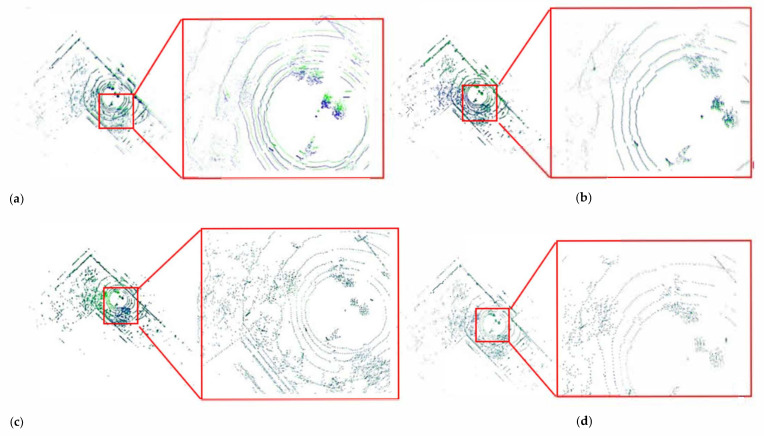
Comparison of registration results of four data association algorithms. (**a**–**d**) are the results of two point clouds aligned respectively by using the NDT algorithm, the ICP algorithm, the FPFH algorithm, the Super-4PCS algorithm.

In Figure 7, there are two point clouds of the adjacent frames, which are marked separately in blue and green. They were aligned by using the ICP algorithm, the FPFH algorithm, the Super-4PCS algorithm and the NDT algorithm, and the registration results are shown from Figure 7a–d, respectively. The translation errors, rotation errors and computing time of the point cloud registration are listed in Table 3.

It can be seen from Table 3, the NDT algorithm took the shortest time, but the rotation error and the translation error were the largest. The Super4PCS algorithm had the smallest rotation error and translation error, but the algorithm took the longest time.

### 3.3. Determine the Appropriate Frame Number of Each Submap

**Figure 8:**
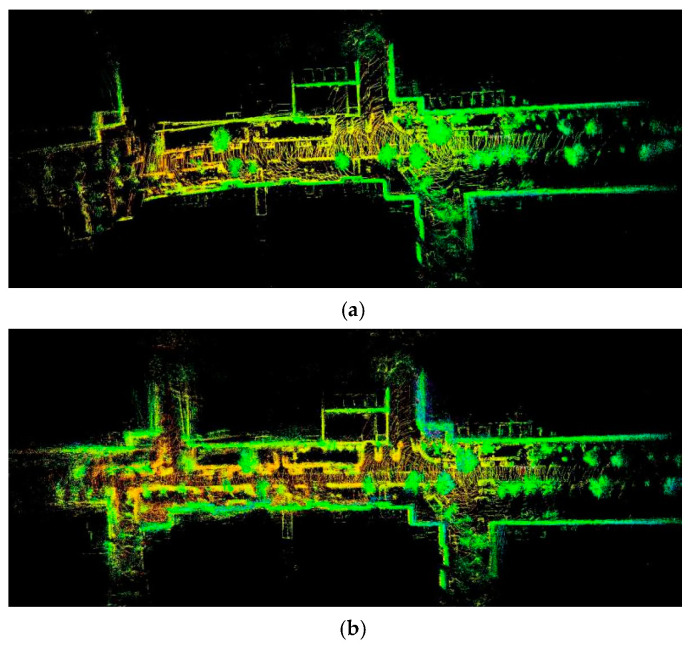

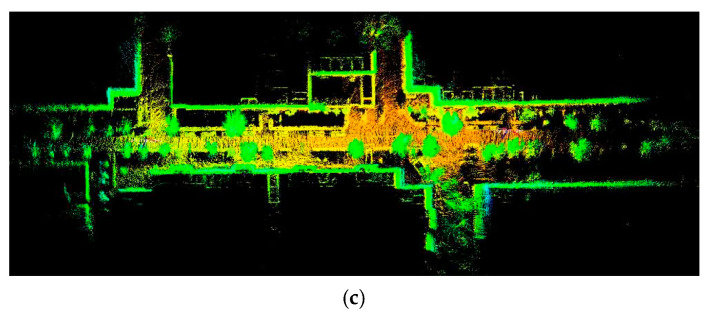
The number of laser frames contained in each submap. (**a**) The submap only contained one laser scan frame; (**b**) the submap only contained three laser scan frames and (**c**) the submap only contained five laser scan frames.

In order to determine the number of laser frames contained in each submap, several experiments were designed and conducted. In the first experiment, the NDT algorithm was applied to match the point clouds between consecutive frames, but the ICP algorithm was not used to associate the submaps when each submap included only one laser frame. The experiment result was shown in Figure 8a, some misalignments appeared in the 3D point cloud map. In the second experiment, each submap included three laser frames, the point clouds between consecutive frames were aligned by using the NDT algorithm, and the key frames between consecutive submaps were matched by using the ICP algorithm. However, there were some misalignments in the 3D point cloud map (Figure 8b). In the third experiment, each submap included five laser frames, there were few misalignments in the 3D point cloud map (Figure 8c). In the fourth experiments, a line segment was selected as shown in Figure 8c, the relative errors were computed between the measurements from the 3D point cloud map constructed by the different submaps with *n* (*n* is from 1 to 6) frames and the truth value, and then the relative errors are shown in Table 4. 

It can be seen from Table 4 that when the frame number *n* of each submap was 1 (*n* = 1), the maximum relative error of the point cloud map was 7.86%. With the frame number *n* of each submap increasing, the quality of the point cloud map was significantly improved. However, when the frame number *n* of each submap was greater than 4, the relative error converged, which means the quality of the point cloud map tended to be stable. Therefore, in this study, the appropriate frame number of each submap was chosen to be 4 (*n* = 4) for the ASQWolf system.

### 3.4. The Underground Garage Experiment

The construction area of the underground garage is 12,000 square meters, the ground is flat and there are many structural features. The continuous-time laser frames were collected when we walked in the underground garage with the ASQWolf system. These consecutive laser frames were fed into the ASQWolf system, and they were processed using the method proposed in this study through interframe alignment, submap match, and closed-loop optimization, a three-dimensional point cloud map was finally formed. 

**Figure 9:**
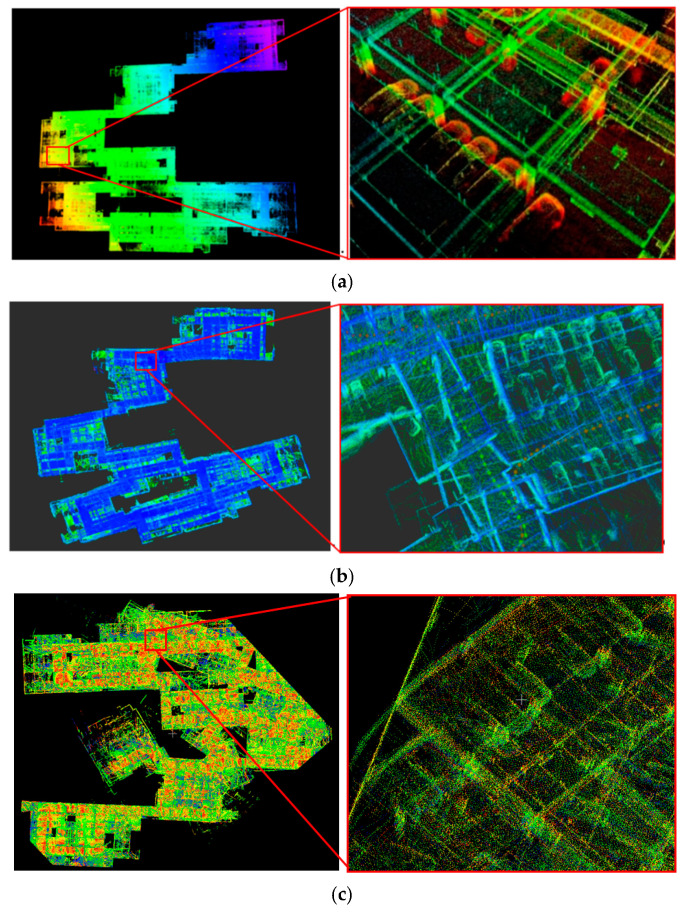
The 3D point cloud map reconstructed by three different algorithms. (**a**) The 3D point cloud map is constructed by our algorithm; (**b**) the result is obtained by the traditional optimization algorithm with interframe alignment and closed-loop optimization and (**c**) the result is obtained by the algorithm with interframe point cloud association and subgraph association.

Figure 9 shows the 3D point cloud maps reconstructed by three different algorithms. From Figure 9a, the structured feature edges in the 3D point cloud map constructed by our algorithm were very clear, without ghosting, and the angle features and the straight-line features were kept. Where the NDT algorithm was only applied to match the consecutive frames, and the ICP algorithm was applied to match the submaps. In Figure 9b, the result was obtained only by a traditional optimization algorithm with interframe alignment and closed-loop optimization and without submap match, the structural features in the environment were not clear, positioning deviation occurs to leading to the generation of point cloud ghosting in the map. Where the NDT algorithm was only applied to match the consecutive frames. Figure 9c was the result of only interframe point cloud association and submap association without closed-loop optimization. Not only the structural features in the environment were not clear, but also due to the large cumulative error, the 3D point cloud map could not be built. 

### 3.5. The Park Environment Experiment

**Figure 10:**
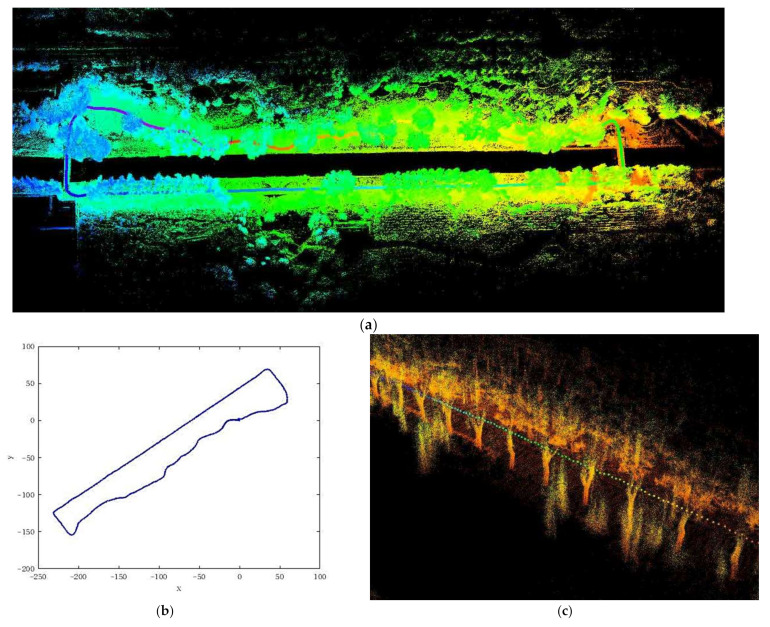

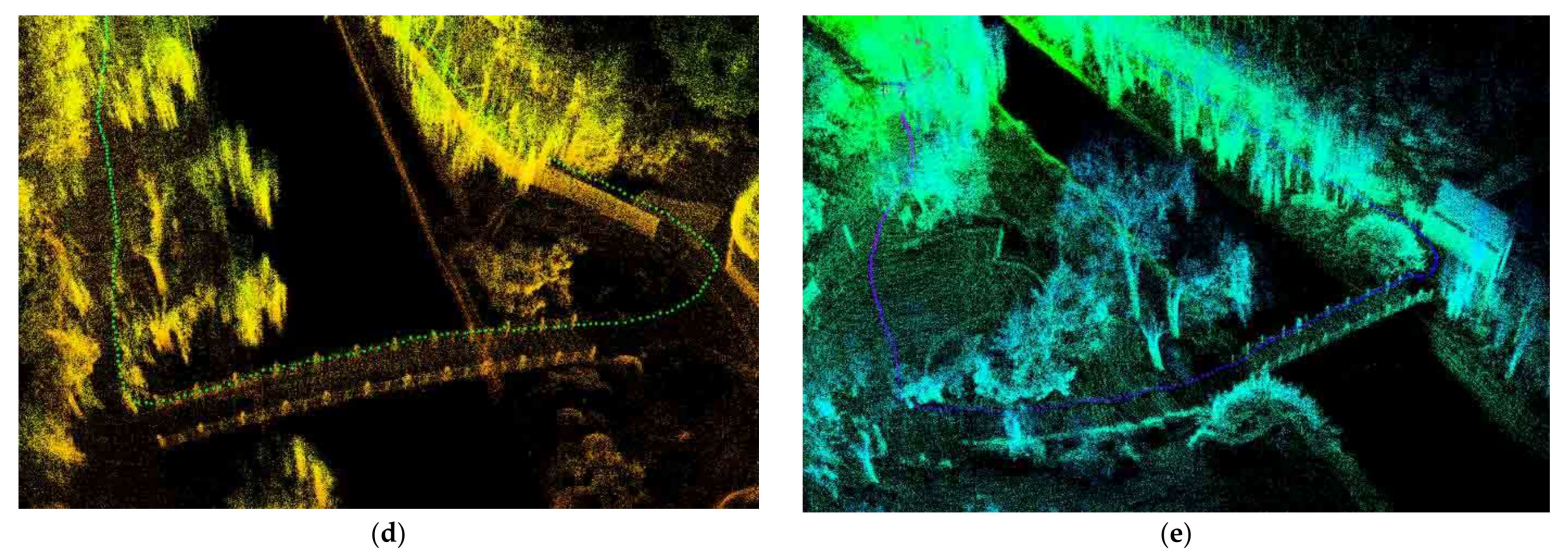
The 3D point cloud map of Yuandu Heritage Park. The ASQWolf system started from the arch bridge (**d**) via the sections of (**c**) and (**e**), and finally returned to the arch bridge (**d**); (**a**) is the 3D point cloud map and (**b**) is the trajectory of the ASQWolf system.

There are many differences between outdoor scenes and indoor scenes as there are a lot of disturbance factors, outliers and unstructured features in the outdoor scene with the uneven ground. We took the ASQWolf system and walked around the river of Yuandu Heritage Park in Haidian District of Beijing, while the ASQWolf system was capturing laser frames at the same time. The laser frames were processed by the method proposed in this study, a 3D point cloud map was generated and shown in Figure 10a. The curve in Figure 10b is the trajectory of the ASQWolf system. The ASQWolf system started from the position of the arch bridge in Figure 10d to collect laser frames, via the sections of Figure 10c,e, and finally returned to the arch bridge shown in Figure 10d The whole trajectory includes up and down slopes, pavements and arch bridges and the total length was about 1.0 km.

The position and attitude of the ASQWolf system at the start point was set as $v_{strat}=\left[0,0,0,0,0,0\right]$, when the ASQWolf system returned to the start point, the position and attitude of the ASQWolf system was $v_{end}=\left[x,y,z,roll,pitch,yaw\right]$, the rotation errors and translation errors of the ASQWolf system were listed in Table 3 by comparing $v_{end}$ and $v_{start}$.

It can be seen from Table 5 that the multilayer pose graph optimization algorithm has higher positioning accuracy than the ordinary pose graph optimization algorithm and algorithms without optimization.

## 4. Discussion

This study mainly focused on the continuous-time laser frames association without other sensors’ auxiliary information. Laser odometry and mapping (LOAM) [22] and lightweight and ground-optimized Lidar odometry and mapping (LeGO-LOAM) [20] applied the structural features to match between consecutive point clouds. When the structural features extracted from point clouds are insufficient in semistructured and unstructured environments, the interframe association is not accurate, even the 3D point cloud map could not be built. The Hdl-graph-slam algorithm [19] used the NDT method to avoid from the structural features, but it is not suitable for unstructured environments to adjust the pose of the point cloud with the ground plane information. There are also many algorithms that used GPS/IMU to improve the SLAM mapping accuracy [29], but the goal of this study is how to associate consecutive laser frames without other sensors’ auxiliary information. The unique advantages and characteristics of the algorithm proposed in this paper are as follows:

(1) Point cloud segmentation: the point cloud of each frame contains ground points and non-ground points. If all the points of each frame participate in the point cloud association calculation, large computation increases the matching time. In this paper, the point cloud of each frame was divided into ground points and non-ground points, and the non-ground points were clustered again to remove the small point clusters (noise point clusters) for reducing interference targets such as leaves and grass. The ground points and non-ground points (except some noise point clusters) of consecutive frames were input into the interframe association layer to be executed the point cloud registration calculation through the NDT algorithm. Considering the amount of calculation, only the non-ground points (except some noise point clusters) of consecutive key frames were aligned between submaps by the ICP algorithm.
(2) Use the NDT algorithm to fast associate laser frames and form submaps. In Section 3.2, although the rotation and translation errors of the NDT algorithm were larger than the ICP, FPFH and Super4PCS algorithms, it was fastest and did not reply on features to associate laser frames, the NDT algorithm based on probability statistics was more reliable, stable and suitable for the association between laser frames with sparse point clouds than the ICP, FPFH and Super4PCS algorithms.
(3) Use the ICP algorithm to match submaps. All consecutive laser frames were aggregated to generate consecutive local submaps of the traversed environment. Each submap accumulated in a short period of time was accurate and consistent enough, the key frames of the submaps could provide a good initial value for the ICP algorithm to match the submaps, avoiding the local minimum of the ICP algorithm. In Section 3.4, the experiment also shows that the NDT algorithm and ICP algorithm cooperated with each other to enhance the robustness and adaptability of the algorithm. The NDT algorithm could fast form the submaps, the ICP algorithm could better align the submaps.
(4) Multilayer graph optimization. Through the first layer interframe association and the second layer submap matching, the association errors were gradually reduced. The submap matching was performed on the basis of the interframe association, which was a further optimization of the association results. The experiments in Section 3.4 showed that the smoothness and consistency of the 3D point cloud map was improved by the multilayer graph optimization proposed in this study; the submap matching is a key step to keep the consistency of continuous-time laser frame associating and mapping. Compared to the result without the submap matching, the proposed method realizes low drift localization and point cloud map reconstruction without any other sensors. The complex associating and mapping problem is modeled as a hierarchical optimization problem.

However, there are many significant improvements still needed to be made before it becomes a mature application. In the future, semantic information will be integrated to perceive the complex dynamic environments, remove moving objects and improve the robustness of the registration approach. In addition, the submap length determination and the key frame selection need to study the more appropriate methods.

## 5. Conclusions

This research successfully completes the accurate association of continuous-time laser frames step by step to build the 3D point cloud map through three levels of interframe association, submap matching and closed-loop optimization. The NDT algorithm and ICP algorithm cooperate with each other to enhance the robustness and adaptability of the algorithm. Through automatic extraction of ground points and automatic segmentation of non-ground points, the point clusters that interfere with the interframe association of the point cloud and the matching of subgraphs were removed. The experimental results show that the method proposed in this study was suitable for underground, indoor and outdoor environments, without the help of GPS, IMU and any other sensors, to realize low drift localization and point cloud map construction.

## Figures and Tables

**Table 1:** Ground initial seed point selection algorithm.

$\mathrm{Input}:\;\mathrm{Original}\;\mathrm{Point}\;\mathrm{Cloud}\;P,\;\mathrm{Initial}\;\mathrm{Seed}\;\mathrm{Number}\;N_{av}\;\mathrm{Output}:\;\mathrm{Selected}\;\mathrm{Ground}\;\mathrm{Initial}\;\mathrm{Seed}\;\mathrm{Point}\;\mathrm{Set}\;$
According to the z-coordinate of each point $p_{i}^{height}$, all points of the point cloud are sorted from smallest to largest to get the ordered point cloud $P_{sorted}$ Take first $N_{av}$ points from the ordered point cloud $P_{sorted}\;$and calculate the mean value $Z_{av}$ of their Z coordinates Compare the size of $p_{i}^{height}$ and $Z_{av}$ for each point $p_{i}\in P$, if $p_{i}^{height}<Z_{av}$, the point is added to the ground initial seed set $P_{seed}$, otherwise, discard the point When all the points of the original point cloud P are compared, stop.

**Table 2:** Ground point extraction algorithm.

$\mathrm{Input}:\;\mathrm{Original}\;\mathrm{Point}\;\mathrm{Cloud}\;\mathrm{Data}\;P,\;\mathrm{Ground}\;\mathrm{Initial}\;\mathrm{Seed}\;\mathrm{Set}\;P_{seed},\;\mathrm{Distance}\;\mathrm{Threshold}\;Th_{dist}\;\;\;\;\;\;\;\;\;\;\;\;\;\;\;\;\;\;\;\;\;\;\;\;\;\;\;\;\;\;\;\;\;\mathrm{Output}:\;\mathrm{Ground}\;\mathrm{Point}\;P_{g},\;\mathrm{Non}-\mathrm{Ground}\;\mathrm{Point}\;P_{ng}$
The ground plane is fitted by singular value decomposition (SVD) from the selected initial seed $P_{seed}$ Calculate the orthogonal distance $Th_{i}\;$ from the point $p_{i}\in P$ to the ground plane fitted in step 1 For point $p_{i}\in P$, if $Th_{i}<Th_{dist}$, the point $p_{i}\;$ is marked as a ground point $P_{g}$; Otherwise, it is marked as a non-ground point $P_{ng}$ Terminate when all points in the point cloud P are marked

**Table 3:** Registration results of four data association algorithms.

Data Association Algorithm	Point Number	Rotation Error (°)	Translation Error (m)	Time (ms)
NDT	25236	0.75	0.809	77
ICP	25236	0.47	0.172	292
FPFH	25236	0.59	0.387	259
Super4PCS	25236	0.24	0.057	1982

**Table 4:** The number of point cloud frames contained in the submap, *n*, for the map quality evaluation in terms of relative error.

Submap Point Cloud Frame Number *n*	Relative Error (%)
1	7.86
2	4.24
3	2.05
4	1.65
5	1.64
6	1.65

**Table 5:** Relative pose error.

Algorithm	Roll	Pitch	Yaw	Total Rot. (°)	x	y	z	Total Trans.(m)
Multilayer Graph Optimization	0.012	0.162	0.320	0.359	0.210	0.214	0.022	0.372
Traditional Pose Graph Optimization	0.032	0.131	0.472	0.491	3.563	3.747	0.836	5.242
Without Optimization	2.783	0.567	2.891	4.053	18.428	14.074	2.851	23.362

## Data Availability

Data sharing not applicable.
